# Intracerebroventricular administration of the (1→6)-β-d-glucan (lasiodiplodan) in male rats prevents d-penicillamine-induced behavioral alterations and lipoperoxidation in the cortex

**DOI:** 10.1080/13880209.2017.1299767

**Published:** 2017-03-08

**Authors:** Carlos Ricardo Maneck Malfatti, Fabio Seidel dos Santos, Jéssica Wouk, Luiz Augusto da Silva, Renan Garcia Michel, André Luiz Snak, Tiago Czervinski, Mário A. Alves da Cunha, Aneli M. Barbosa, Robert F. H. Dekker

**Affiliations:** a Pharmaceutical Science Postgraduate Program, Midwest State University, Campus CEDETEG, Guarapuava, Brazil;; b Teaching of Science and Technology Postgraduate Program, Federal Technological University of Paraná, Campus Ponta Grossa, Ponta Grossa, Brazil;; c Department of Chemistry, Federal Technological University of Paraná, Campus Pato branco, Pato Branco, Brazil;; d Department of Chemistry, Londrina State University, Londrina, Brazil;; e Environmental Engineering Postgraduate Program, Federal Technological University of Paraná, Campus Londrina, Londrina, Brazil

**Keywords:** Convulsions, free radicals, GABAergic system, neurotoxicity

## Abstract

**Context:** Lasiodiplodan, an exocellular (1→6)-β-d-glucan of molecular weight >1.4 × 10^6^ Da produced by MMPI strain of *Lasiodiplodia theobromae* (Pat.) Griffon & Maubl. (Brotyosphaeriaceae) is known to exhibit anti-proliferative activity on breast cancer cells (MCF-7), anticoagulant activity when sulfonylated, and reduction in transaminase activity when administered in rats.

**Objective:** The effect of intracerebroventricular (I.C.V) injection of lasiodiplodan on neurotoxicity and behavioural changes induced by d-penicillamine was investigated.

**Materials and methods:** Twenty-four male Wistar rats were initially separated in groups of six and treated with 0.15 μmol/μL of NaCl (Groups Ct and d-Pen) and 0.01 μg/μL of lasiodiplodan (Groups Las and Las + d-Pen). After 15 min, they received 6 μmol/μL of NaCl (Groups Ct and Las) and 2 μmol/μL of d-penicillamine (Groups d-Pen and Las + d-Pen). The animal behavior was observed in an open-field test for 60 min. Twenty-four h later, the animals were sacrificed and histopathological analysis and Thiobarbituric acid reactive substances (TBARS) production measurements were performed.

**Results:** Lasiodiplodan prevented neurotoxicity induced by d-penicillamine significantly reducing the production of TBARS (308%; *p* < 0.05), and behavioural signs; convulsive and pre-convulsive. No histopathological alterations in the cerebral cortex were observed.

**Discussion and conclusion:** The reduction of TBARS production and convulsive episodes suggests that the protector effect provided by lasiodiplodan passes thought an antioxidant path, possibly interfering in a cascade of neurochemical events, triggering cell death and convulsive episodes. These results demonstrated that lasiodiplodan can be effective in treating neurotoxicity, and reducing damage triggered by convulsions in neuropathies related to GABAergic system.

## Introduction

d-Penicillamine, a potent copper-chelating agent, is used in treating Wilson’s disease (hepatolenticular degeneration), an autosomal recessive genetic disorder caused by mutations in the *ATP7B* gene which results in abnormal copper metabolism and subsequent accumulation of copper in tissues, especially, the liver and brain (Lorincz 2010). Neurological symptoms of this disease include variable combinations of dysarthria, dystonia, tremor and choreoathetosis. Penicillamine also demonstrated positive effects in treating other pathological conditions such as rheumatoid arthritis and cystinuria (Walshe 2011).

Vitamin B_6_ antagonist drugs, like d-penicillamine and hydrazine, however, inhibit the activity of enzymes such as glutamic acid decarboxylase (GAD), glutamine synthetase, and GABA transaminase (GABA-T), and induce inhibition of mitochondrial activity in some encephalic regions of the brain. The inhibition of this group of enzymes leads to a decrease in gamma-aminobutyric acid (GABA) concentration, which predisposes convulsive episodes (Abe 1978; Abe & Matsuda 1979) while the drugs that increase GABAergic activity, like the benzodiazepines, and GABAa and GABAb agonists (muscimol and baclofen, respectively), presents anticonvulsant action (Malfatti et al. 2007). Besides this, d-penicillamine has been related to a rise of reactive oxygen species (ROS) in response to oxidative stress that leads to the formation of thiobarbituric acid-reactive substances (TBARS, including lipid hydroperoxides) in the rat’s cerebral cortex (Ciuffi et al. 1992; Chen et al. 2012), with the inhibition of key enzymes in the Krebs cycle (which are dependent on sulfhydryl groups) leading to neuronal cell death (Walshe 2011).

Much interest has been generated on antioxidants because of their protection role against several chronic pathologies that involve excessive production of ROS, including cancer, cardiovascular and neurodegenerative diseases, such as Alzheimer disease (AD), Parkinson disease (PD) and amyotrophic lateral sclerosis (ALS) (Gaté et al. 1999; Emerit et al. 2004). These substances act reducing behavioural and neurochemical manifestations related to neurotoxicity, epileptogenesis and neuronal cell death (Martinc et al. 2012).

The fungal β-d-glucans and their derivatives exhibit Biological Response Modifying (BRM) activities, which depend upon their structure, molecular weight and degree of substitution (Synytsya & Novák 2013). β-d-Glucans have demonstrated biological activities, including anti-inflammation, anticoagulation, antithrombosis, antioxidation, anticancer, antitumor, antiviral, hypoglycaemic, hypocholesterolemic activities (Kagimura et al. 2015a). The exopolysaccharide employed in this study was lasiodiplodan, a (1→ 6)-β-d-glucan with triple helix structure (Vasconcelos et al. 2008) obtained from the MMPI fungal strain of *Lasiodiplodia theobromae* (Pat.) Griffon & Maubl. (Brotyosphaeriaceae) when grown on glucose. Infrared and magnetic resonance analysis were recently performed to confirm the chemical structure of this substance (Kagimura et al. 2015b). Studies related to these β-glucans are considered rare, and the few studies reported show that lasiodiplodan presents antiproliferative activity in breast cancer (MCF-7) cells (Cunha et al. 2012), anticoagulant activity when sulfonylated (Vasconcelos et al. 2013), antioxidant activity, which is enhanced when the biomolecule is carboxymethylated (Kagimura et al. 2015b) well as hypoglycaemic activity and reduction of transaminase activity in rats without any hematologic and histologic changes that indicate toxicity in the vital organs (Túrmina et al. 2012). Lasiodiplodan has not yet been tested in experimental and clinical neurotoxicity models. Therefore, considering this background, the objective of the work reported here was to analyze the effect of lasiodiplodan administered by I.C.V injection in rats on the neurotoxicity and behavioural changes induced by d-penicillamine in the central nervous system.

## Materials and methods

### Animals

This study included 24 male 60-day-old Wistar rats. The animals were maintained in cages (4 animals per cage) under controlled conditions of temperature (26 ± 1 °C), light-dark cycle of 12/12 h, and *ad libitum* access to water and feed (PURINA^®^). All experimental procedures in this study were approved by the Institutional Ethics Committee on Animal Use (protocol no. 089/2013). Efforts were made to minimize animal suffering, as well as to reduce the number of animals needed for this study.

### Experimental design

Rats were randomly assigned into four groups each of 6 animals: control (Ct), lasiodiplodan (Las), d-Penicillamine (d-Pen), lasiodiplodan + d-Penicillamine (Las + d-Pen). d-Penicillamine was purchased from Sigma (St. Louis, MO).

### Lasiodiplodan production and evaluation of molecular weight

Lasiodiplodan was produced by *Lasiodiplodia theobromae* MMPI in submerged fermentation using a 2 L benchtop fermenter (Biostat B, B. Braun International, Germany) operated at a temperature of 28 °C, agitation of 400 rpm, air-flow set at 0.8 vvm and an initial pH of the nutrient medium of 5.5. The fermenter vessel contained 1 L of nutrient medium consisting of minimum salts medium (Vogel 1956) and glucose (20 g/L), and was inoculated with 100 mL of the inoculum prepared as described by Cunha et al. (2012). After 72 h of cultivation, the fermentation was interrupted and the fungal biomass was separated by centrifugation (1500 × *g*, 30 min). The supernatant containing the lasiodiplodan was recovered and exhaustively dialyzed against cold water (4 °C) using dialysis membranes (≅12,000 Da, 1.3 in. width, MWCO 11,331, Sigma-Aldrich, USA). Lasiodiplodan was precipitated from the dialyzed supernatant with 3 vol. abs. ethanol, and left standing at 4 °C overnight. The precipitate was recovered by filtration, and re-solubilized in distilled water at 60 °C under agitation. The resulting solution was exhaustively dialyzed against distilled water and then lyophilized.

The molecular weight (MW) of lasiodiplodan was estimated by gel permeation chromatography in a High Performance Size Exclusion Chromatograph (HPSEC; Shimadzu, Model RID 10 A, Japan) using dextran as molecular weight standards. Lasiodiplodan solution (1 mg of total sugar/mL) was filtered through a Millipore membrane (cellulose acetate) with 0.22 μM porosity. Aliquots of 200 μL were injected into the chromatograph equipped with differential refractive index detector (model RID 10 A). Four gel permeation columns (Waters, USA) with excluding limits of 7 × 10^6^, 4 × 10^5^, 8 × 10^4^ and 5 × 10^3^ Da arranged in series, were employed. Sodium nitrate solution (0.1 M) containing sodium azide (0.003%, w/v) was used as the mobile phase with a flow rate of 0.6 mL/min, and operated under a pressure of 9.8 MPa and temperature of 37 °C. Dextran molecular weight standards between 14 × 10^6^ g/mol to 9.4 × 10^3^ g/mol were used to construct a calibration curve, which allowed to estimate the molecular weight of lasiodiplodan.

### Stereotaxic protocol

Animals were anesthetized intraperitoneally with a mixture of xylazine hydrochloride (5 to 10 mg/kg) and ketamine hydrochloride (50 to 75 mg/kg). Anesthetized animals were placed in a rodent stereotaxic apparatus (Insight^®^, ETX 3/99, Ribeirão Preto) for the unilateral insertion of a cannula (28 gauge, length 8 mm) in into the right lateral ventricle of the brain following the bregma coordinates, using a set of three coordinates (x, y, and z) (in mm): AP, 0; L, 1.5; V, 3.0 from the dura (Paxinos & Watson 1986). The cannula was secured with acrylic resin. Chloramphenicol (200 mg/kg i.p.) was administered immediately before the surgical procedure.

### Microinfusion

Microinfusion was initiated at least 72 h after surgery. Initially, the animals were injected (I.C.V.) with 2 μL of a solution of the following concentrations: 0.15 μmol/μL of NaCl (Groups Ct and d-Pen) or 0.01 μg/μL of lasiodiplodan (Groups Las and Las + d-Pen), using the guide (27 gauge, length 9 mm). After 15 min, the animals were injected with 6 μmol/μL of NaCl (Groups Ct and Las) or 2 μmol/μL of d-penicillamine (Groups d-Pen and Las + d-Pen). The dose and volume of lasiodiplodan injection (0.01 μg/μL) was chosen according to the maximum concentration at which the polysaccharide was soluble in water and of fluid consistency, so as not to impede flow through the cannula, which avoided cannula reflux in the animals, and allowed observation at the site of injection. d-Penicillamine was injected with a similar dosage (2 μmol/μL) as reported by Gross et al. (1994).

### Behaviour observation

An open field was used to analyze animal behaviour in response to the drugs tested. The apparatus consisted of a 54.7 cm diameter acrylic box that permits observation of the animal’s behaviour. After the experimental treatments, each animal was gently placed in the open field and allowed to explore the arena for a period of 60 min. The primary objective of this procedure was to identify clonic or tonic convulsions. The following behaviours were also measured: time spent convulsing, tail extensions, wet-dog shakes, self-grooming and rearing. The results were expressed in seconds (s). This task was videotaped to monitor the behaviour of the rats following administration of d-Pen and Las + d-Pen.

### Morphological parameters

Rats of each study group were sacrificed by decapitation 24 h after the last behavioural session, during which they were fasted for 12 h. For histological analyzes, the cerebral cortex was collected. Initially, this tissue was fixed in 10% formaldehyde for 24 h, and then transferred to a stock solution (ethyl alcohol, 70% v/v) and stored until required for histological analysis. Afterwards, the tissues were embedded in paraffin and sectioned using a microtome, and the slices stained with haematoxylin-eosin (H.E).

The cerebral cortex tissues were examined for any histopathological changes. Pathological diagnostic of each tissue specimen was assessed and analyzed by a histopathologist.

### Lipid peroxidation measurement

Lipid peroxidation was determined by measuring thiobarbituric acid-reactive substances (TBARS) quantified by the reaction between malondialdehyde (MDA) and thiobarbituric acid (TBA); a well-established method for quantifying lipid peroxides (Devasagayam et al. 2003). Samples of the brain were collected and homogenized in 50 mM sodium phosphate buffer (pH 7.4). The homogenate samples (250 μL) were then mixed with 1 mL 10% (w/v) trichloroacetic acid (TCA) and 1 mL of 0.67% (w/v) thiobarbituric acid, and the resulting mixtures vortexed, and then heated at 98 °C for 15 min, followed by cooling in ice water. After this step, 2 mL of *n-*butanol was added, and the samples vortexed for another 1 min. The mixtures were then centrifuged (1200 × *g* for 5 min), and the absorbance of the samples measured at 535 nm using a spectrophotometer.

### Statistical analyses

All results are presented as the mean ± S.E.M. Statistical analysis was performed using one-way ANOVA. Differences were considered statistically significant at *p <* 0.05. The *post hoc* Bonferroni’s Multiple Comparison was used to identify differences between the groups when appropriate.

## Results

### Molecular weight of lasiodiplodan

The calibration curve obtained by plotting the molecular weight (Log MW) of dextran standards versus retention times (Log MW = −0.16373 X + 13.17564) presented by an adequate correlation coefficient (*R*^2^ = 0.98605), and allowed the estimation of the molecular weight of lasiodiplodan to be >1.4 × 10^6^ Da.

### Behavioural observations

The results of behavioural observations as viewed on the videotapes recorded showed that i.c.v. injection with a solution of d-penicillamine (2 μmol/μL) caused the appearance of convulsions in the d-Pen Group, characterized by episodes of myoclonic jerks involving forelimbs and hindlimbs [F_3.20_ = 4.90, *p* = 0.01], and by a significant increase of the other behavioural manifestations typical of tonic-clonic convulsions, such as tail extensions [F_3.20_ = 6.83, *p* = 0.02] and stereotyped movements (wet-dog shakes) of the animals [F_3.20_ = 3.50, *p* = 0.03]. Administered lasiodiplodan was found to be capable of reverting all of these mentioned behavioural characteristics.

The results also demonstrated that both d-penicillamine and lasiodiplodan affected the animal’s exploratory behaviour. The rearing time was significantly lower in the d-Pen group and higher in the Las group compared to the control [F_3.20_ = 88.03, *p* = 0.0001]. No significant difference was observed on the duration of self-grooming among the experimental groups [F_3.20_ = 1.36, *p* = 0.28]. The results of behavioural observation are presented in Table 1.

**Table 1.** Effects of d-penicillamine and lasiodiplodan on behavioral parameters.

	Groups			
Parameters (s)	Ct	Las	d-Pen	Las + d-Pen
Time spent convulsing	0.0 ± 0.0	0.0 ± 0.0	280.0 ± 0.0^a^	0.0 ± 0.0
Wet dog-shake	0.0 ± 0.0	0.0 ± 0.0	210.0 ± 94.0^a^	50.0 ± 50.0
Rearing	570.0 ± 33.76^b^	940.0 ± 50.60^a^	280.0 ± 25.3	250.0 ± 18.44
Self-grooming	140.0 ± 25.3	150.0 ± 25.7	220.0 ± 40.0	170.0 ± 28.6
Tail extension	160.0 ± 36.8	260.0 ± 30.0	340.0 ± 42.9^c^	150.0 ± 25.7

Data are expressed as mean ± S.E.M. Superscripts (a,b,c) represent statistical differences of *p < *0.05, one-way ANOVA with *post-hoc Bonferroni’s Multiple Comparison* (*n* = 6) animals/group.

a Significantly different from others groups.

b Significantly different from the d-Pen and Las + d-Pen groups.

c Significantly different from the Ct and Las + d-Pen groups.

### Lipid peroxidation measurement

**Figure 1.**
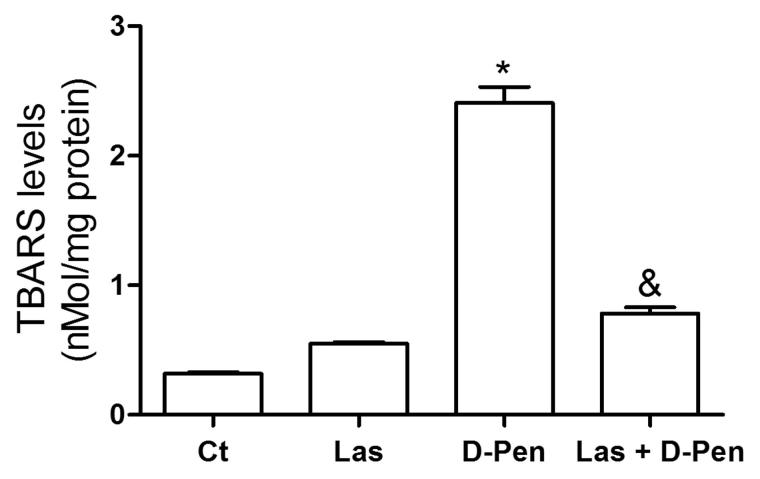
Effects of D-penicillamine and lasiodiplodan on TBARS levels. Data are expressed as mean ± S.E.M. *Statistical difference between the D-Pen group and the other groups (*p < *0.05); ^&^Statistical difference between the Las + D-Pen group and Ct (*p < *0.05) - (one-way ANOVA with *post-hoc Bonferroni’s Multiple Comparison test, n* = 6 animals/group).

The results showed that d-penicillamine administration significantly increased TBARS levels (750%) in the cerebral cortex of the rats (d-Pen: 2.36 ± 0.11 vs. Ct: 0.32 ± 0.01; *p < *0.05). Lasiodiplodan administration was able to attenuate this effect, reducing TBARS levels around 308% [Las + d-Pen: 0.80 ± 0.05 vs. d-Pen 2.36 ± 0.12; *p* < 0.05; F_3.20_ = 196.8, *p < *0.05]. The results of TBARS levels are shown in Figure 1.

### Histopathological analysis

**Figure 2.**
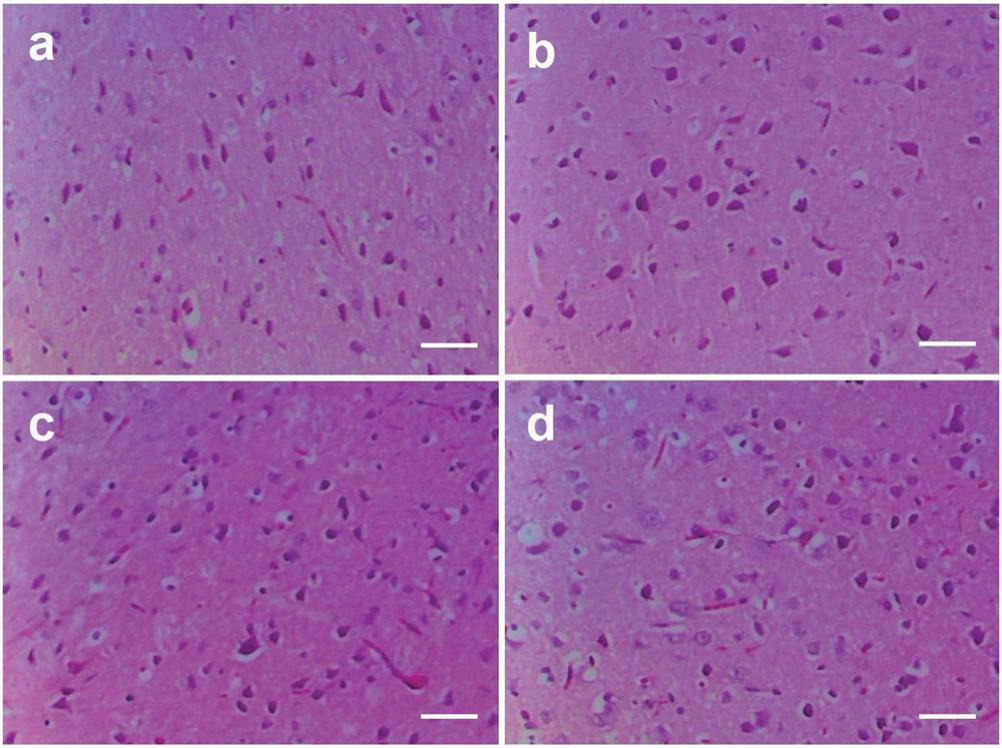
Effects of D-penicillamine and lasiodiplodan on cerebral cortex histology of rats (100x). (a) cerebral cortex from the Ct group; (b) cerebral cortex from the Las group; (c) cerebral cortex from the D-Pen group; (d) cerebral cortex from Las + D-Pen group. *Scale bar* 25 μm.

No histopathological alterations in the cerebral cortex of the rats among the experimental groups were observed (Figure 2).

## Discussion

The effects of (1→6)-β-d-glucan (lasiodiplodan) on the central nervous system have not hitherto been described in the scientific literature. Other activities of biotechnological interest, and particularly those in the health field, have been described for lasiodiplodan: absence of toxicity under sub-chronic usage (Túrmina et al. 2012), antiproliferative effect in breast cancer MCF-7 cells (Cunha et al. 2012), an effect recently reported to be associated with oxidative stress, necrosis, and apoptosis induced by AMP-activated protein-kinase (AMPK) and the Forkhead transcription factor, FOXO3a (Queiroz et al. 2015).

In this study we demonstrated that I.C.V injection-of lasiodiplodan in male rats prevented pre-convulsion behavioral signs, with typical automatism of tonic-clonic convulsions (wet-dog shakes and tail extensions), and additionally, it prevented damage resulting from free radicals as quantified by TBARS production in the brain cortex. d-Penicillamine is an antagonist drug of vitamin B_6_ (pyridoxine) that affects the enzymes, GAD and GABA-T, leading to a decrease in GABA concentration predisposing convulsive episodes (Abe 1978; Abe & Matsuda 1979). Pyridoxine is fundamental to GABA’s function, and the deficiency of this substance occurs in pathological conditions like pyridoxine-dependent epilepsy, convulsions that can be controlled by administration of vitamin B_6_ (Rajesh & Girija 2003).

Literature data shows the involvement of ROS after administration of d-penicillamine in lab animals, which facilitates the formation of TBARS and lipid hydroperoxides in the cortex of the rat’s brain (Ciuffi et al. 1992; Chen et al. 2012). ROS also prejudices the function of the GABAergic system, and enhances excitability and convulsions (Malfatti et al. 2007) that can be one of the mechanisms on how GABAergic antagonists generate convulsions (Oliveira et al. 2004). It is important to emphasize that GAD is highly sensitive to free radicals, and its inhibition amplifies epileptogenesis induced by FeCl_3_ (Robitaille et al. 1995). In this way, it is probable that the antioxidant activity of lasiodiplodan (Giese et al. 2015; Kagimura et al. 2015a, 2015b) has contributed to attenuate the neurotoxic effects of d-penicillamine. It is well-known that non-cellulosic β-glucans present antioxidant activity, scavenging ability of free radicals and block lipid peroxidation (Kayali et al. 2005; Kofuji et al. 2012). Studies have shown that β-glucans present neuro-protective effects against neurotoxicity induced by free radicals in the brain and sciatic nerves of diabetic rats, and in animals with spinal cord injury (Kayali et al. 2005; Alp et al. 2012).

The molecular weight can have a significant influence on the physicochemical characteristics and biological properties of the biomolecules (Zhang et al. 2013). Du and Xu (2014) observed that the molecular weight and the source of β-glucans were intimately related to their antioxidant potential. These researchers noticed that β-glucans with high molecular weights, for example, yeast carboxymethyl-β-glucan (404 kDa) and oat (1→3)(1→4)-β-glucan (409 kDa), displayed the strongest antioxidant activity, determined by the ferric reducing antioxidant power (FRAP) (Du & Xu 2014).

It has been demonstrated that (1→6)-β-d-glucan (lasiodiplodan) has capacity for scavenging the ABTS-radical cation, as well as the ability to scavenge DPPH-radicals, proving its antioxidant activity (Kagimura et al. 2015b). Although the antioxidant power of this polysaccharide has been proven by many biochemical test previously mentioned, further works are necessary to elucidate the possible antioxidant mechanism of β-glucan.

In this way, the administration of lasiodiplodan in the central nervous system could possibly be effective in providing great protection against the deleterious effect (damage) of GAD inhibition and the neurochemical consequences that trigger convulsive episodes with exacerbated production of reactive oxygen species (ROS) and cell death. In this work, we verified that the lasiodiplodan provided a total reversion of the convulsive episodes caused by the selective inhibitor of GAD administration; this effect may also be correlated with the significant reduction of TBARS production. The inverse relation between TBARS production and convulsive episodes suggests that the protector effect provided by lasiodiplodan passes thought an antioxidant path, possibly interfering in a cascade of neurochemical events that trigger cell death and convulsive episodes, such as, free radicals attack to biological membrane; selectivity loss in these membranes with a higher influx of sodium and calcium (Shin et al. 2008; Stringer & Xu 2008; Engel & Henshall 2009); rise of nitric oxide synthase activity (Royes et al. 2007; Chen et al. 2008); mitochondrial death; and neuronal energetic deficit trigging a failure in pumps (Na^+^, K^+^-ATPase activity) that are important to the neuronal repolarization (Waldbaum & Patel 2010) causing the exacerbation of electric discharges (seizures) observed by convulsive behavior and electroencephalographic alterations in the animals (Malfatti et al. 2007; Ribeiro et al. 2009).

## Conclusions

In a general way, the results of this study have indicated that lasiodiplodan prevented signals of neurotoxicity induced by d-penicillamine, attenuating significantly lipid peroxidation in the brain cortex, as well as typical automatism of convulsions. These results suggest that lasiodiplodan can be effective in the prevention of neurotoxicity, as well as attenuating damage provoked by the convulsive episodes related to the GABAergic system.
